# Supplementary material for: A Critical View of the Application of the APEX Software (Aqueous Photochemistry of Environmentally-Occurring Xenobiotics) to Predict Photoreaction Kinetics in Surface Freshwaters
Source: Molecules. 2019 Dec 18;25(1):9. doi: 10.3390/molecules25010009 (PMC7017383; doi:10.3390/molecules25010009)
Supplement: Supplementary file 1 [file molecules-25-00009-s001.zip › 3.2.4_gcc-4.4.0.zip!/3.2.4_gcc-4.4.0/3.2.4_gcc-4.4.0/doc/octave/html/interpreter/Operator-Index.html]

Operator Index - Untitled


Previous: Function Index,
Up: Top


---

## Operator Index

- !: Element-by-element Boolean Operators
- !=: Comparison Ops
- ": String Objects
- ": Strings
- &: Element-by-element Boolean Operators
- &&: Short-circuit Boolean Operators
- ': String Objects
- ': Arithmetic Ops
- ': Strings
- (: Index Expressions
- ): Index Expressions
- \*: Arithmetic Ops
- \*\*: Arithmetic Ops
- \*=: Assignment Ops
- +: Arithmetic Ops
- ++: Increment Ops
- +=: Assignment Ops
- ,: Matrices
- -: Arithmetic Ops
- `--`: Increment Ops
- -=: Assignment Ops
- .': Arithmetic Ops
- .\*: Arithmetic Ops
- .\*\*: Arithmetic Ops
- .+: Arithmetic Ops
- ./: Arithmetic Ops
- .\: Arithmetic Ops
- .^: Arithmetic Ops
- /: Arithmetic Ops
- /=: Assignment Ops
- ;: Matrices
- <: Comparison Ops
- <=: Comparison Ops
- =: Assignment Ops
- ==: Comparison Ops
- >: Comparison Ops
- >=: Comparison Ops
- [: Matrices
- \: Arithmetic Ops
- ]: Matrices
- ^: Arithmetic Ops
- colon: Ranges
- |: Element-by-element Boolean Operators
- ||: Short-circuit Boolean Operators
- ~: Element-by-element Boolean Operators
- ~=: Comparison Ops
